# Sunflower sea star predation on urchins can facilitate kelp forest recovery

**DOI:** 10.1098/rspb.2022.1897

**Published:** 2023-02-22

**Authors:** A. W. E. Galloway, S. A. Gravem, J. N. Kobelt, W. N. Heady, D. K. Okamoto, D. M. Sivitilli, V. R. Saccomanno, J. Hodin, R. Whippo

**Affiliations:** ^1^ Oregon Institute of Marine Biology, Department of Biology, University of Oregon, 63466 Boat Basin Road, Charleston OR 97420, USA; ^2^ Department of Integrative Biology and Partnership for Interdisciplinary Studies of Coastal Oceans, Oregon State University, 3029 Cordley Hall, Corvallis, OR 97331, USA; ^3^ School of Aquatic and Fishery Sciences, University of Washington, 98195, Seattle WA, USA; ^4^ Astrobiology Program, University of Washington, 98195, Seattle WA, USA; ^5^ Department of Psychology, University of Washington, 98195, Seattle WA, USA; ^6^ Friday Harbor Laboratories, University of Washington, 98195, Seattle WA, USA; ^7^ The Nature Conservancy, Sacramento CA, 95811, USA; ^8^ Department of Biological Science, Florida State University, Tallahassee, 32306 FL, USA

**Keywords:** sunflower sea stars, *Pycnopodia helianthoides*, purple sea urchins, *Strongylocentrotus purpuratus*, kelp forest ecology, predation

## Abstract

The recent collapse of predatory sunflower sea stars (*Pycnopodia helianthoides*) owing to sea star wasting disease (SSWD) is hypothesized to have contributed to proliferation of sea urchin barrens and losses of kelp forests on the North American west coast. We used experiments and a model to test whether restored *Pycnopodia* populations may help recover kelp forests through their consumption of nutritionally poor purple sea urchins (*Strongylocentrotus purpuratus*) typical of barrens. *Pycnopodia* consumed 0.68 *S. purpuratus* d^−1^, and our model and sensitivity analysis shows that the magnitude of recent *Pycnopodia* declines is consistent with urchin proliferation after modest sea urchin recruitment, and even small *Pycnopodia* recoveries could generally lead to lower densities of sea urchins that are consistent with kelp-urchin coexistence. *Pycnopodia* seem unable to chemically distinguish starved from fed urchins and indeed have higher predation rates on starved urchins owing to shorter handling times. These results highlight the importance of *Pycnopodia* in regulating purple sea urchin populations and maintaining healthy kelp forests through top-down control. The recovery of this important predator to densities commonly found prior to SSWD, whether through natural means or human-assisted reintroductions, may therefore be a key step in kelp forest restoration at ecologically significant scales.

## 1. Introduction

When predators have a significant influence on the populations or behaviour of herbivores, they can exert top-down control into food webs [1]. Strong predators of herbivores can trigger a trophic cascade, providing an indirect benefit to primary producers by reducing grazing rates of herbivorous consumers [2,3]. The strength of a predator can be owing to direct consumption of its prey [4] (a consumptive effect), or through behavioural changes in the prey owing to the ‘landscape of fear’ that the predator imparts on the system [5]. The details of this relationship are also governed by the hunger level of the predator and its herbivorous prey and the productivity at the base of the food chain [3]. Importantly, the strength of a trophic cascade may weaken when the prey are hungry. First, particularly hungry herbivores may be more likely to engage in risky foraging behaviour, even when the predator is nearby, which weakens the cascading effect [6,7]. Second, predators may be less likely to hunt or consume hungry prey because they may be less nutritious [8]. This weakening of a trophic cascade is especially problematic when a herbivore population has become overpopulated and is causing harm to its ecosystem via overgrazing, which often occurs after a predator decline. In this case, reintroduction or recovery of a predator may not have the intended cascading effect because prey do not stop foraging or the predator does not pursue the low-quality prey.

The sunflower star (*Pycnopodia helianthoides*) is a broadly distributed generalist predator, which exploits diverse intertidal and subtidal habitats, from Alaska to Baja California Mexico. *Pycnopodia* is one of the largest sea stars in the world [9], reaching greater than 1 m in diameter. *Pycnopodia* are highly mobile, and they use their speed and size to hunt a large variety of invertebrates, including bivalves, gastropods, echinoderms, crabs and carrion of any origin [9,10]. Because of their diverse diets, speed and prowess as predators, *Pycnopodia* are in a position to exert significant top-down control on benthic communities; indeed their declines are often correlated with kelp forest collapse [11–13]. *Pycnopodia* are eager predators of herbivorous purple sea urchins *Strongylocentrotus purpuratus* [14–16], but their predation rates and potential impacts on these urchins in the field are still unknown.

Populations of *Pycnopodia* and other sea stars have been decimated across their range in the northeast Pacific ocean by a sea star wasting disease (SSWD) which emerged in 2013 [17]. *Pycnopodia* have declined by greater than 97% from historic population levels on the Pacific coast from Washington through to Baja California, have shown few signs of recovery, and are now considered critically endangered [10,18]. The loss of *Pycnopodia* in parts of British Columbia and California has contributed to drastic change in the community composition of kelp forests; herbivorous grazers, especially *S. purpuratus*, have increased dramatically presumably owing to the lack of top-down control by *Pycnopodia* [11–13,19]. The ecosystem consequences of SSWD are intertwined with a major northeast Pacific marine heatwave that persisted from 2014 to 2016, resulting in greater than 90% reduction in the canopy forming bull kelp *Nereocystis luetkeana* in the near shore waters of the northern-central California coast [12,13].

Declines of bull kelp forests are concerning for ecosystem functioning, because kelp are highly productive, habitat forming foundation taxa that support a myriad of species [20], which are often considered to be primary conservation targets, such as abalone, sea otters and rockfish. Kelp forests are dynamic ecosystems [21] that experience booms and busts at decadal time scales [22,23], governed both by ocean conditions and by grazers such as sea urchins and other herbivores [24]. Kelp forest invertebrate communities can be surprisingly resilient to heatwaves [25], but the background variation of dynamic kelp communities makes it difficult to assess the importance of specific consumers in these systems. In healthy and productive kelp forests, sea urchin diets are supported by an abundance of drifting kelp detritus; but if kelp standing stock declines, sea urchins increasingly graze directly on established and juvenile seaweeds [26], which can shift the ecosystem state to a ‘barren’ alternative stable state [27]. Sea urchins can dominate and persist in these barrens for long periods because they are able to regulate their metabolism and survive in a near-starvation state for years [28,29].

Owing to the persistence of sea urchins once they are established, and their ability to control kelp forests, there is growing interest throughout the west coast of North America to understand how sea urchin mortality [30] and removal efforts [31] may encourage kelp forest recovery. Sea urchin culling efforts may create important community engagement and small changes to the reefs where the work is occurring [31], but these localized interventions can only affect kelp forests at a minimal spatial scale. Encouraging the recovery of natural sea urchin predators may be the most effective path towards successful kelp forest recovery. Sea otters (*Enhydra lutris*) were probably the most dominant predator of sea urchins throughout the northeast Pacific before they were largely extirpated in the fur trade [32]. Sea otters are recovering in southeast Alaska, British Columbia, and parts of Washington and California, but these discerning predators generally avoid hunting sea urchins in barrens because urchins in these habitats have little gonad content and are therefore less nutritious [8]. However, it has been shown in the Alaskan Aleutian Islands that while individual sea urchins in barrens have lower caloric content, the high densities of urchins in barrens can make the per unit area content of calories available in urchins similar to that of kelp forests [33]. In areas where sea otters have re-colonized islands with barrens, those areas have reverted back to kelp forests [33]. Other important sea urchin predators include the California sheephead (*Semicossyphus pulcher*) and spiny lobster (*Panulirus interruptus*) [34,35], but these species are found only in southern California and southwards, and therefore do not benefit northern kelp forests.

The geographically widespread sudden collapse of kelp forests and dramatic increases in sea urchin abundance just after the *Pycnopodia* decline is striking and supports decades-old field experiments [15] that suggest this sea star predator may actually be a critically important component of healthy kelp forests ecosystems. Because of this, there is growing interest in management action to reintroduce the species along much of the United States west coast, and a captive breeding programme is underway [36]. There is concern that efforts to restore kelp forests by reintroducing *Pycnopodia* will be thwarted because the sea stars will simply avoid consuming sea urchins in barrens and switch to higher quality prey elsewhere. To determine whether *Pycnopodia* recovery and possible reintroduction is a viable tool to help restore kelp forest ecosystems, more information about the consumptive effects of sea stars on nutritionally poor sea urchins in barrens is needed.

We investigated the potential role of *Pycnopodia* as a predator of *S. purpuratus* in different nutritional states that mimic the low energetic value of sea urchins found in barrens and the high energetic value of well-fed and gonad-rich urchins that are found in kelp forests. We focused on *S. purpuratus* because of the growing concern on the North American west coast in northern and central California and Oregon about growing *S. purpuratus* urchin barrens. We first used a conditioning protocol to create analogues of urchin barren and kelp forest sea urchins. We then performed experiments using sunflower sea stars and the two classes of conditioned urchins to address the questions: (i) do *Pycnopodia* have a preference for fed sea urchins?; (ii) how many sea urchins can *Pycnopodia* eat per day?; (iii) is *Pycnopodia* predation rate on sea urchins affected by whether the urchins are themselves fed or starved?; and (iv) does *Pycnopodia* hunting behaviour and *S. purpuratus* defensive or fleeing behaviour differ for fed or starved urchins? We then used a population model parameterized with these data to investigate the consequences of *Pycnopodia* reintroduction in west coast sea urchin barrens, and the potential benefits to kelp forest communities.

## 2. Methods

### (a) Study area and *Pycnopodia* collections

We performed the collections and experiments in the San Juan Islands, Washington, at Friday Harbor Laboratories (FHL) in the summer of 2020. FHL has access to remnant populations of *Pycnopodia*, which is now a rarity. Collections were made with the approval of the director of FHL under the auspices of Washington state statute with FHL as the managing agency.

We collected a total of 24 individual *Pycnopodia*, in a size range of 30–52 cm diameter (41.2 ± 7.1 cm, mean ± s.d.), over the course of 15 dives at seven locations. *Pycnopodia* were rare at all sites; but on average, we captured two stars per bottom-time hour. Divers recorded the site characteristics for every collected *Pycnopodia* including whether it came from an area known to have populations of *S. purpuratus* for later analysis (see below). Because of the threat of SSWD, we treated all *Pycnopodia* with exceptional care, minimizing stressful handling time. After collection, all animals were returned to FHL immediately and transferred to four dedicated holding tanks. Individual *Pycnopodia* vary in size, number of arms, overall coloration and sometimes in specific patterns of coloration (electronic supplementary material, figure S1). We used these unique patterns and characteristics to name and study the natural history and variation in behaviour among individuals [36]. During their four months in captivity, we fed the *Pycnopodia* a ‘maintenance diet’ of two mussels (*Mytilus edulis*) per star every 2 days when they were not in other predation trial protocols. The methods used for *Pycnopodia* housing, prey and feeding frequency were informed by Hodin *et al*. [36]. The general experimental schedule for all sea stars, including experimental group numbers, timelines for all experiments, and metadata on all individual stars used in the experiment are presented in the electronic supplementary material, tables S1 and S2.

### (b) Sea urchin collections and conditioning

*Strongylocentrotus purpuratus* is very rare in the San Juan Islands, but can be found in some shallow locations (0–5 m depth) in the southern and western areas of San Juan Island (A.W.E. Galloway 2010-2022, personal observations). *S. purpuratus* does not currently form urchin barrens in this system. We therefore used a conditioning protocol to create urchin barren analogue urchins by starving and spawning out urchins, and kelp forest analogue urchins by feeding the urchins with kelp ad libitum prior to the predation trials (e.g. [37]), summarized in the electronic supplementary material, table S1.

We conducted three collection dives over 18–20 July 2020 at sites with known populations of *S. purpuratus* near Cattle Pass, between Lopez and San Juan Islands. We collected 300 *S. purpuratus* from rocky subtidal habitat with healthy bull kelp forests and high flow, in roughly the interquartile range of the sizes we observed in the field, ranging from 45 to 70 mm in diameter (57.9 ± 5.3, mean ± s.d.). Sea urchins were transferred to the laboratory after capture and sorted into two shaded long-term holding tanks with an equal number of urchins of roughly the same test diameter size distribution. We randomly assigned each tank to the feeding or starvation treatment. On 20 July 2020, we started to feed the urchins in the feeding treatment tank bull kelp (*N. luetkeana*) blades, and we did not feed urchins in the starvation treatment tank. On 30 July (10–12 days after collection), we sacrificed 20 new urchins collected from the same urchin collection site to assess gonad index from ‘wild’ urchins for comparison with our experimental urchins.

From 15 to 17 August 2020 (approx. 1 month after collection), we induced spawning in the urchins in the starvation treatment, in order to further diminish gonadal condition. Urchins were injected through the peristomal membrane with 0.05 M KCl at 0.02 ml of KCl per 1 ml urchin volume. In total, 53% of starving urchins were successfully spawned (gamete release observed) and of the urchins that spawned, 27% were female and 73% were male, indicating that many of the urchins that did not spawn were non-gravid females. All injected urchins were allowed to recover in holding tanks for at least 19 days before the start of any sea star experiment, and urchins continued to starve during the recovery period.

Gonadal condition was assessed several times throughout the experiment to measure changes to gonad through time: 30 July (*n* = 13, ‘wild’); 7–10 September (*n* = 15 ‘pre-experiment starved’ and *n* = 14 ‘pre-experiment fed’) and 22–23 October (*n* = 9 ‘post-experiment starved’ and *n* = 15 ‘post-experiment fed’). Whole urchin weight (g), test diameter (mm) and height (mm) were recorded prior to dissection. Urchins were dissected along the equator and all internal tissues were carefully removed. Digesta was removed from the gut, and tissues were blotted for 30 s prior to weighing. Gonad wet weight (g) in addition to gut plus gonad weight (g) were recorded. Gonadal index was calculated as 100*[gonad weight/urchin volume in cm^3^]. Urchin volume was calculated using the formula for an oblate spheroid as [4/3π * radius^2^ * (0.5*height)]. We documented a significant difference in gonadal condition between fed and starved urchins within 40 days and this difference was maintained throughout the experiment days (electronic supplementary material, figure S2 and table S3). To determine if our conditioning treatments were indeed reflective of the body condition of sea urchins in kelp forests and urchin barrens, we compared the gonad indices of our starved and fed treatments to published gonadal index data [37] from the Channel Islands, California during the spawning season (December–March) of 2011–2014. We analysed the differences in gonad index between fed, starved, kelp forest and urchin barren urchins using a generalized linear model (*stats* package) and follow-up tests (*lsmeans* package) in R v.4.0.0 [38] and RStudio 1.2.5042.

### (c) *Pycnopodia* prey choice experiment

We performed prey choice experiments between 14 and 29 October 2020 (electronic supplementary material, table S1) on each star to assess whether *Pycnopodia* could detect chemical cues and exhibit a preference for nutritionally valuable fed sea urchins typical of a kelp forest over starved sea urchins typical of an urchin barren. We used a large plexiglass y-maze (57 cm width × 170 cm length) in two types of y-maze trials: (i) one side of the maze having no urchin, the other having several fed or starved urchins (to assess whether stars could identify any prey in the experiment); and (ii) one side of the maze with fed urchins and the other with starved sea urchins (to assess whether the stars selected fed or starved urchins). Urchins in all trials were held behind a perforated barrier at the top of the maze which did not allow the urchins to move or the *Pycnopodia* to capture them. We ran each of the 24 stars through each y-maze trial once, and all stars had previous experience with purple sea urchins, since the choice experiments were performed following the predation rate experiments (below). Flow rates on each side of the y-maze were adjusted until they were equal by measuring total water volume dispensed over time (0.34 l s^−1^). For each replicate trial, the star was released at the base of the maze, and the run was scored as a ‘choice’ for the prey if the sea star advanced up the y-maze 90 cm. Occasionally the stars did not move from the start of the maze, and the run was scored as ‘no choice’ and was not included in analysis. To minimize side bias, water flow rate in each side of the y-maze was equalized before the start of each run, and the treatments were alternated between left and right sides of the y-maze. Total runs were *n* = 19 and *n* = 21 for trial types 1 and 2, respectively (*n*'s are < 24 owing to cases where stars made ‘no choice’ as described above). To determine if one choice was more likely than the other, we ran a chi-squared test for each trial, using *chisq.test()* in the *stats* package in R [38].

### (d) *Pycnopodia* predation rate experiment

We set up predation experiments as 6–7 day trials, buffered by a 24 h acclimation period after transferring a star to a new location before the start of any trial (see the electronic supplementary material, tables S1 and S2 for dates and assignments of all individual stars to all treatments). We used 12 replicate aquaria (60 cm wide × 90 cm long × 30 cm deep) with an inflow of seawater on one side and the outflow on the opposite end. We covered the tanks with two large outdoor tents so they received no direct sunlight, but were still exposed to indirect ambient light and with a natural light cycle. We conducted a total of four trials using 12 tanks and sea stars each, such that each of the 24 sea stars were used in two of the four 6–7-day trials, with the sea urchin treatment assigned to a given star switching between trials (see the electronic supplementary material, figure S4). In between trials, sea star hunger levels were reset by resting in their holding tank for 6 days with their maintenance diet (described above), followed by 5 days of starvation.

At the start of each trial, two sea urchins (either both fed or both starved, always from the same feeding treatment) were added to the tank. We checked on the tanks every 12 h, recorded the status of the urchins (captured or egested) and if an urchin had been captured within that time frame, we would add a new urchin from the same treatment into that tank. No star consumed both urchins in any 12 h period, thus, there was always at least one live sea urchin in the tank with each *Pycnopodia*, ensuring that they could feed ad libitum. At the end of each trial, we moved the stars back to their long-term holding tanks and scrubbed the aquaria with fresh water to remove chemical cues from the previous trial.

We calculated predation rates as urchins captured per day, handling times as the hours elapsed between capture and egestion, and ‘rest’ times as the hours elapsed between egestion of one sea urchin and capture of another. Note that we checked the tanks only every 12 h, so while the rates and time estimates may be somewhat imprecise, our high replication allows us to accurately estimate these values, and that the imprecision should not affect our comparisons between treatments. We analysed *Pycnopodia* predation rates, handling times and rest times using a mixed effect linear model in JMP Pro 16 (SAS Institute) fitted using restricted estimated maximum likelihood. We tested the effects of urchin treatment (fed versus starved) and *Pycnopodia* source habitat (purple urchins present or absent) and their interaction on the number of urchins captured per day in each tank. We included the trial and the sea star as random variables to control for the non-independence of tanks tested in the same trial and of sea stars being used in successive trials. We also investigated whether sea star and urchin diameter affected predation rate, handing time and rest time, and found no relationships. We also tested whether *Pycnopodia* collected from areas with and without *S. purpuratus* differed in their consumption rates of fed versus starved urchins.

### (e) Quantifying *Pycnopodia* and urchin movement and behaviour

Predation trials were fully video-recorded during daylight hours with GoPro HERO5 cameras mounted facing down and directly above each of the 12 aquaria. We used the videos to measure animal movements and several interactions between the predator and prey. Urchin and sea star position over the course of each trial were tracked using DeepLabCut pose estimation software [39], a Python toolbox that employs deep neural networks to track animal features. We trained two networks on a subset of still video frames (approx. 300 frames each) to recognize the objects of interest: network 1 tracked the corners of the experimental tank and the centre of the sea star; network 2 tracked the centre of each sea urchin within the tank. We visually overlaid the urchin, seastar and tank *x, y* coordinates with the raw video footage in Python to confirm the quality of the networks' tracking. Additional frames were labelled and networks were retrained as needed to improve tracking performance. We filtered out videos with technical problems or poor lighting conditions and used the pose estimation software to analyse a total of 298 daily videos, which included 11 stars from trial 1, 11 stars from trial 2, 10 stars from trial 3 and 10 stars from trial 4. Video data from a 10 h daytime period (8.00–21.00) when all experimental tanks were well-lit and tracking was optimal was used for a subset of days from each trial (days 2–7 for trial 1, days 2–6 for trials 2–4). Timestamped coordinate data from the urchin and sea star networks were merged into a single dataset to compare sea star and urchin movement patterns through time and comprised 5 843 701 total coordinates between all time-points and species.

To quantify sea urchin and *Pycnopodia* movement, we analysed the movement trajectories for each animal using the as.ltraj function in the *adehabitatLT* package in R. For each time step, we calculated the rate of movement of each animal and the distance between the sea star and each sea urchin. All data were converted from pixels to cm using a ruler in a still frame at the start of each video. We excluded day 7 from experiments since not all trials lasted 7 days, and excluded any speeds greater than 40 cm min^−1^ for *Pycnopodia* and greater than 12 cm min^−1^ for sea urchins since these values were rare and probably owing to video tracking errors. To distill the data into relevant and analysable time frames, we first calculated the average hourly movement of and distance between each animal, then analysed these hourly data. For sea urchins, we averaged the data for the two urchins in a given tank for each hour since the video tracking frequently switched the numbered designations between individuals (i.e. urchin 1 did not necessarily represent the same urchin for the duration of the video nor trial). We used mixed effects generalized linear models (*lme* package) in R to test the effects of sea urchin treatment, day of the experiment, and their interaction on the hourly movement of sea urchins, included tank number as random variable to control for repeated measures, and specified a negative binomial distribution. We performed a similar model on *Pycnopodia* movement, but also included *Pycnopodia* source habitat (*S. purpuratus* present or absent) and its interactions with urchin treatment and day of experiment. We did not include sea urchin sizes in the movement models because video tracking did not always reliably track individual urchins. We investigated whether sea star size affected their movement, but found no patterns.

### (f) Population model

To assess the possible impacts of *Pycnopodia* predation on purple sea urchin density, we used a modelling exercise based on available California and Oregon subtidal community data. Specifically, we asked how *Pycnopodia* density and known handling time can alter mean densities of purple urchins in a simple, heuristic model grounded in empirical field data. We did not use a size-structured model in this case as we focus on predation of mid to-large sea urchins and medium size *Pycnopodia*, which showed little size-dependence in the rate of predator-driven mortality, and thus the numeric effects of a size-structured and size-agnostic model are identical under the assumption that *Pycnopodia* attack rate and handling time of urchins is consistent by urchin size. Importantly, this solution is a conservative estimate, as *Pycnopodia* probably are capable of consuming more small than mid- and large-sized *S. purpuratus* per unit time because of reduced handling time.

The cumulative abundance of urchins can be represented by the following equation:

2.1 $$\frac{dN}{dt}=r-[m+f\;(P(t),N(t))]\;N(t),$$

where *N*(*t*) represents urchin density, *P*(*t*) represents *Pycnopodia* density, *r* represents recruitment rate of urchins (no. of per year entering at less than 2 cm), *m* represents non-*Pycnopodia-*related *per capita* mortality rate (per year), and *Pycnopodia-*related mortality (*f*(*P*(*t*), *N*(*t*)), per prey, per year) is some function of *Pycnopodia* density *P*(*t*), and urchin density *N*(*t*). If we assume *f*(*P*(*t*), *N*(*t*)) follows a Type II functional response (i.e. consumption rate per predator, per prey, per year at high prey densities is limited by handling time) then the equation expands to

2.2 $$\frac{dN}{dt}=r-\left[m+\frac{\alpha P(t)}{1+\alpha hN(t)}\right]\;N(t),$$

where *h* represents the handling time for each *Pycnopodia* per *S. purpuratus* captured while *α* represents the attack rate per *Pycnopodia*, per prey, per year. The functional response asymptotes (at high urchin densities) at 1/*h* urchins per predator per unit time. While this model has no analytical solution (it is a nonlinear function of *N*(*t*)), the expected equilibrium density of urchins is easily solved numerically. The expected density depends on sea urchin recruitment rates (*r*), non-*Pycnopodia*-related mortality (*m*) as well as *Pycnopodia* density (*P*), attack rate (*α*) and handling time (*h*). Because *Pycnopodia* move relatively quickly to capture new prey, and prey are often in high aggregated densities, we assume *α* is high relative to *h*, leading to consumption rates near *h* until *N*(*t*) declines to very low densities. Because the recruitment of *S. purpuratus* can vary dramatically among locations and time periods [40,41], we vary recruitment (*r*) of adult urchins (approx. 2 cm) to illustrate how differences in population productivity affect the control that *Pycnopodia* can exert on urchin population size. We test for sensitivity of the model outputs to the addition of alternative prey consumed by *Pycnopodia* in addition to purple sea urchins. This alternative prey (*S*) has a partial refuge from the predator (i.e. where the second prey item moves freely among a refuge with no behavioural modification from the predator) where the value of the second prey relative to urchins is given by *ρ*. We use a standard multi-resource functional response, where the dynamics of urchins are given by

2.3 $$\frac{dN}{dt}=r-\left[m+\frac{\alpha P(t)}{1+\alpha hN(t)+\alpha \rho hS(t)}\right]\;N(t).$$

The alternative prey is governed by standard logistic growth with carrying capacity *K* and intrinsic growth rate *β* but only 50% of the population is susceptible to predation at any given time, meaning there are always alternative prey available even when predator densities are very high. Because we use a functional response with alternative prey, the realized predation rates at equilibrium will always be lower than the maximum *per capita* rate measured in the laboratory; thus, we also recorded the realized *per capita* predation rate of urchins at equilibrium in the simulations in addition to urchin density. Both the expected densities of *S. purpuratus* and the realized predation rates will depend on preference for alternative prey relative to *S. purpuratus (ρ)* in addition to densities of *Pycnopodia* and urchin recruitment. Thus, we simulate values of *ρ* (0.1, 0.5, 1) that correspond to a *S. purpuratus*: alternative prey preference of 10 : 1, 2 : 1 and 1 : 1. This approach provides a more conservative and realistic translation from the maximum predation rate on sea urchins we measured in the laboratory to an estimated realized predation rate in the field, where predation rates decrease at low prey densities (e.g. increases in search time, accessibility to refuges, etc.) and alternative prey are available to sea stars.

## 3. Results

### (a) Sea urchin conditioning

By the start of the predation trials, after conditioning the sea urchins for 40 d (on 8 September 2020), gonad index (GI) was 2.2 x higher in fed compared to starved urchins (electronic supplementary material, figure S2a and table S3; mean GI ± s.d. starved = 8.39 ± 2.69 and fed = 18.68 ± 6.14). By the end of the trails at 84 d (on 22 October 2020), this difference increased to 3.6 x higher (mean GI ± s.d. starved: 7.79 ± 3.86 and fed 27.92 ± 4.01). When comparing our conditioned animals to wild urchins collected in the San Juan islands at the start of the experiment (mean GI ± s.d. = 16.37 ± 4.47), the wild urchins were intermediate in gonad weight to experimental starved and fed urchins. This indicates that our experimental conditioning was effective in both increasing gonad weights of fed urchins and decreasing the weights in starved urchins. We used the differences in gonad size between urchin treatments to examine *Pycnopodia* response to starved and fed urchins in subsequent experiments. The effectiveness of our diet conditioning was more pronounced in larger compared to smaller sea urchins (electronic supplementary material, figure S2b and table S3). A comparison of the GIs of conditioned sea urchins to wild urchins from kelp forests in urchin barrens in southern California (data from [37]) showed that fed urchins had higher GI than those from kelp forests, and starved urchins had intermediate GI compared to urchins in barrens and kelp forests, (electronic supplementary material, figure S5; *p* < 0.001 for all comparisons).

### (b) *Pycnopodia* prey choice experiment

In y-maze choice trials, *Pycnopodia* chose to pursue a sea urchin more often than no prey (66.6% and 20.9% of trials, respectively) (electronic supplementary material, figure S6a; *χ*^2^ = 5.76, *p* = 0.016). *Pycnopodia* did not show a preference between fed (42.7% of trials) and starved urchins (37.5% of trials) (electronic supplementary material, figure S6b; *χ*^2^ = 0.05, *p* = 0.819). *Pycnopodia* made no choice between a sea urchin and no prey in 12.5% of trials and between fed and starved urchins in 20.8% of trials.

### (c) *Pycnopodia* predation rate experiment

**Figure 1.**
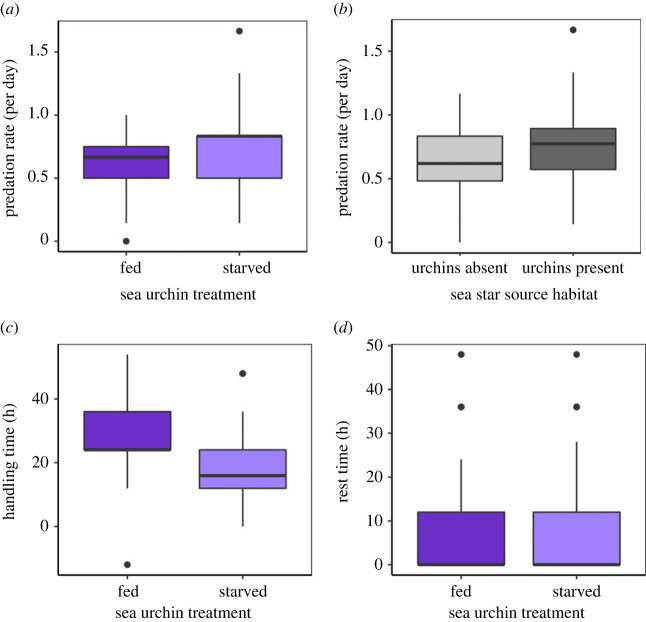
Box plots of predation rates (*a*,*b*), handling time ((*c*); time between capture and egestion) and rest time ((*d*); time between egestion and capture of another urchin) by *Pycnopodia helianthoides* during ad libitum laboratory predation trials on the purple sea urchin *Strongylocentrotus purpuratus.* In (*a*,*c*,*d*), dark purple are well-fed urchins (mimicking kelp forest urchins), and light purple are experimentally starved urchins (mimicking *S. purpuratus* from urchin barrens). (*b*) The influence of the *Pycnopodia*'s source habitat (either without (light grey) or with (dark grey) *S. purpuratus* present) on its predation rate on *S. purpuratus*.

On average, *Pycnopodia* consumed 0.68 ± 0.33 (mean ± s.d.; lower 95% confidence interval (CI) = 0.59, upper 95% CI = 0.78) urchins individual^−1^ d^−1^, across all trials (figure 1*a*). *Pycnopodia* consumed significantly more (21% increase; electronic supplementary material, table S4a; *p* = 0.024) starved than fed sea urchins per day (figure 1*a*; mean ± s.d.: 0.74 ± 0.38 and 0.61 ± 0.25 starved and fed urchins individual^−1^ d^−1^, respectively). Further, we found that *Pycnopodia* which were collected from habitats with *S. purpuratus* present ate 33% more urchins (electronic supplementary material, table S4a; *p* = 0.026) than *Pycnopodia* that were presumably unfamiliar with this prey species (figure 1*b*; mean ± s.d.: 0.80 ± 0.35 and 0.60 ± 0.29 urchins individual^−1^ d^−1^, respectively). *Pycnopodia* handling times (time between capture and egestion) were longer for fed than starved sea urchins (figure 1*c*; electronic supplementary material, table S4b; mean ± s.d.: 25.4 ± 10.5 and 18.4 ± 7.8 h, respectively), but their rest times between meals did not differ with urchin conditioning treatment (figure 1*d*; electronic supplementary material, table S4c; mean ± s.d.: 9.0 ± 12.1 and 8.1 ± 11.1 h, respectively).

### (d) *Pycnopodia* and urchin movement and behaviour

**Figure 2.**
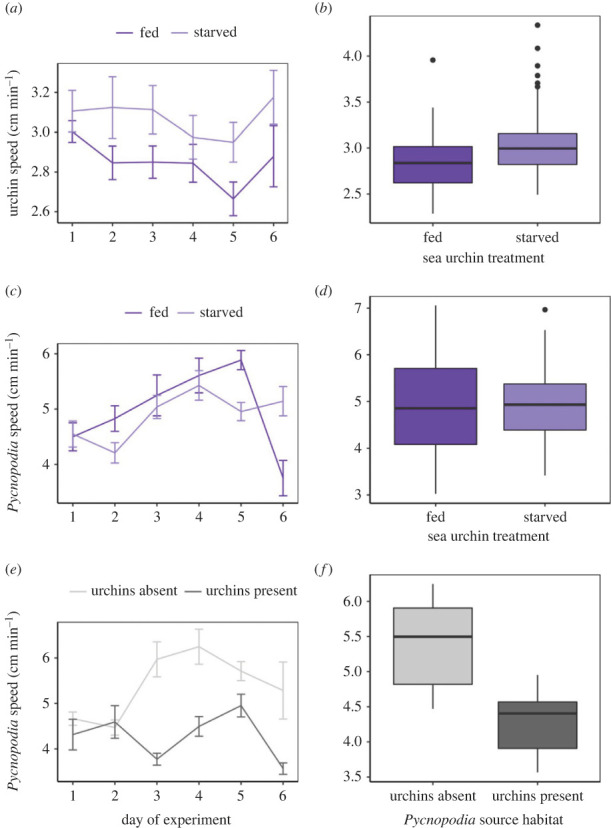
Speeds of animals over time (*a*,*c*,*e*) and on average (*b*,*d*,*f*) estimated as daily average speed in cm per minute from time lapse videos during *Pycnopodia* predation trials on *S. purpuratus*. Colours follow figure 1 caption. (*a*,*b*) Fed versus starved sea urchin speed when exposed to *Pycnopodia*. (*c*,*d*) *Pycnopodia* movement in the presence of these same types of sea urchins. (*e*,*f*) *Pycnopodia* movement depending on its source habitat: *S. purpuratus* present or absent.

Overall, when placed in the aquarium with *Pycnopodia*, the speed of starved sea urchins tended to be higher than the speed of fed sea urchins across all trials (figure 2*a,b*; mean ± s.d.: 3.07 ± 0.38 and 2.85 ± 0.31 cm min^−1^, respectively). Though this difference was not significant (*p* = 0.061), it was consistent for the duration of the trials (figure 2*a*). On the other hand, the average distance between the sea urchins and the sea stars did not differ with sea urchin treatment nor the day of the trial. The speed of *Pycnopodia* did not differ depending on whether they were in the tank with fed or starved urchins (figure 2*c*,*d*), and follow-up tests on the significant day * urchin treatment interaction (figure 2*c*) showed no pairwise differences in *Pycnopodia* speed depending on urchin treatment on any day. However, *Pycnopodia* from sites without purple sea urchins moved more than those from sites with purple sea urchins (figure 2*e*,*f*; mean ± s.d.: 5.39 ± 1.28 and 4.28 ± 0.90, cm min^−1^, respectively), especially during days 3–6 of the trial (figure 2*e*). The statistics for sea star and sea urchin movement are presented in the electronic supplementary material, table S5.

### (e) Population model

**Figure 3.**
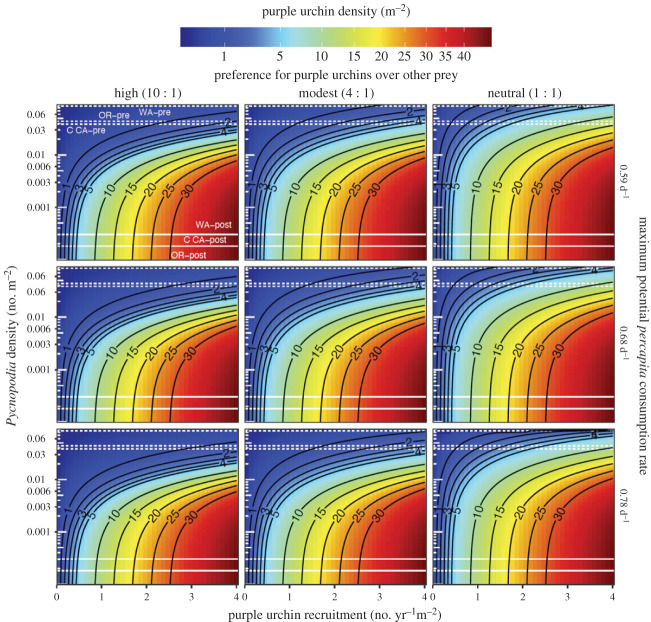
Expected density of adult purple sea urchins from the model as a function of the density of *Pycnopodia* and the recruitment productivity of purple urchins. The columns show different levels of the ‘preference’ of *Pycnopodia* for *S. purpuratus* relative to alternative prey that are highly productive; higher values are greater preference for urchins and are the inverse of *ρ*_alt_ as explained in the Methods. The rows show different levels of maximum potential *per capita* predation rate on *S. purpuratus* by *Pycnopodia* as the mean (0.68 d^−1^, centre row) the upper (0.78 d^−1^, top row) and lower (0.59 d^−1^, bottom row) bounds of the 95% CI for the observed feeding rates in the laboratory experiments. These maximum potential predation rates represent conservative estimates because experimental predation rates are on large urchins, and smaller urchins are probably eaten more quickly. Dotted white lines indicate mean population densities of *Pycnopodia* pre-SSWD collapse, and solid white lines indicate mean post-collapse population densities for the outer coast of Washington (WA), Oregon (OR) and central coast of California (C CA) taken from the International Union for Conservation of Nature data [10]. Note that per capita predation rates (urchins consumed per predator per day) in the simulation will generally be lower than the maximum potential *per capita* consumption rate because predation declines as urchin densities decline via the functional response; the realized predation rates associated with these simulations are shown in the electronic supplementary material, figure S7.

We found that *Pycnopodia* densities prior to population crashes (approx. 0.06 per m^2^) are largely incompatible with high urchin densities (figure 3). Moreover, only very low densities of *Pycnopodia* (approx. less than 0.01 m^2^) allowed for very high urchin densities (greater than 15 m^2^), and such high urchin densities required substantial recruitment (greater than 3.5 m^2^ yr^−1^) to overwhelm even low *Pycnopodia* densities (figure 3). The model indicates that, at a conservative level (i.e. without higher predation on younger size classes), even small recoveries in *Pycnopodia* can lead to far lower expected densities of sea urchins. The model results are generally robust to the predation rate used (i.e. across the 95% CI for consumption from predation rate experiments) in comparison to the impact of variation in *Pycnopodia* densities or urchin recruitment rates. By contrast, expectations of the role of *Pycnopodia* in affecting urchin densities are quite sensitive to *Pycnopodia* foraging preferences and behaviour in nature. Specifically, if *Pycnopodia* consume other highly productive prey with equal preference relative to purple urchins (e.g. figure 3, column 3), then substantially more *Pycnopodia* are required to achieve the same low densities of purple urchins. Importantly, these results illustrate that while maximum potential per capita predation rates in the laboratory are quite high, expected long-term per capita predation rates in the field are much lower (between approx. 0.05 and 0.3 urchins d^−1^ *Pycnopodia*^−1^ at high *Pycnopodia* densities—electronic supplementary material, figure S7). This result occurs because urchin densities are reduced dramatically and *Pycnopodia* are expected to consume other prey in proportion to abundance and preference.

## 4. Discussion

Our laboratory experiments, paired with a population model, provide to our knowledge, the first demonstrable mechanistic evidence that the sunflower sea star *Pycnopodia helianthoides* can potentially impart strong top-down control on purple sea urchin (*S. purpuratus*) populations. In our predation experiments, we found that *Pycnopodia* consumed 0.68 ± 0.33 (mean ± s.d.) *S. purpuratus* per day. The predation rates observed here are approximately 5 x higher than the only other known previous estimate for *Pycnopodia* predation rates of urchins in this size range [15]. However, those estimated predation rates [15] were inferred for green (*Strongylocentrotus droebachiensis*) and purple urchins combined, from field observations, using observations of handling time, proportion of urchins in the diet and proportions of successful attacks. In that work, Duggins [15] found that 68% of feeding *Pycnopodia* in Torch Bay, AK, were feeding on sea urchins, and he concluded that *Pycnopodia* were likely to be important for regulating local urchin populations. Until now, there have been no published studies quantifying the predation rates of *Pycnopodia* on purple sea urchins, and thus no way to reasonably model the direct consumptive effects of this predator on this prey. Our combined experimental and modelling results illustrate that the decline in predation rates by *Pycnopodia* after local extirpation from SSWD in 2013–2016 [10] was enough to release sea urchins from top-down control except at quite low sea urchin recruitment rates.

Overgrazing by sea urchins is a major threat to kelp forests worldwide and a major driver of the collapse of kelp forests [12], along with climate change and marine heatwaves [42,43]. While it has been hypothesized from correlational studies that the near-extirpation of *Pycnopodia* owing to SSWD was a contributing factor in the subsequent kelp collapse, our study suggests it may have been a central driver. Managers are increasingly seeking diverse tools for kelp forest conservation [44], including the reintroduction of captive-raised *Pycnopodia* to reefs [36]. Our work suggests that *Pycnopodia* recovery may be an effective and long-lasting method to control sea urchin populations and restore kelp forest ecosystems.

Prior to this study, it was unknown whether *Pycnopodia* would simply avoid eating the nutrient-poor sea urchins, and thus be ineffective at exerting top-down control in urchin barrens. We found that *Pycnopodia*'s predation rate was actually higher on starved and gonad-depleted urchins compared to well-fed urchins with large gonads (figure 1). This indicates that *Pycnopodia* may even exert stronger top-down control on urchin populations in barrens than in kelp forests. At the very least, they do not appear to avoid eating these less nutritious prey. This increased predation rate on starved urchins differs from the foraging patterns of other urchin predators. The top keystone predator in the system, the sea otter, tends to avoid eating nutrient-poor sea urchins in barrens in favour of foraging on more nutritious urchins elsewhere. In southern California and Baja California, Mexico, *S. purpuratus* are also predated upon by California sheephead and spiny lobster. However, spiny lobsters appear to prefer *S. purpuratus* from kelp forests with larger gonads [45]. As they grow larger, sea urchins increasingly benefit from a size refuge from predators such as California sheephead [46,47]. Overall, our findings suggest that the relative importance of *Pycnopodia* compared to sea otters and other predators may increase when a location is in an urchin barren rather than kelp forest state. By contrast, in areas where *Pycnopodia* and sea otters overlap, *Pycnopodia* may serve as a complementary secondary predator [11] to the much more voracious sea otter. This agrees with prior research which shows *Pycnopodia* can create herbivore-free areas that benefit kelp assemblages, even in areas with high sea otter density [15].

There are several mechanisms that may be driving the higher predation rates on starved than fed urchins. Our y-maze experiment was designed to test whether *Pycnopodia* chemically sense starved *S. purpuratus,* and choose to pursue these potentially weaker prey, as other studies have shown that *Pycnopodia* prefer damaged prey relative to healthy individuals [48]. However, while *Pycnopodia* clearly identified *S. purpuratus* as a potential prey, they did not prefer either fed or starved urchins. This suggests that the *Pycnopodia* are not actively seeking these starved and probably weaker barren urchins, nor are they avoiding them. Instead, shorter handling times for starved urchins may be driving the higher predation rates on starved urchins because they take less time to consume. It did not appear that *Pycnopodia* rested more between meals when urchins were well-fed, indicating that they did not become satiated during the experiments. Further, rest times were often short; it was common for *Pycnopodia* to capture more than one urchin at a time, or for them to egest and capture a new urchin between our 12 h observation increments. Other possible mechanisms driving *Pycnopodia's* higher predation rates on starved urchins are the hunting behaviours of the sea stars or the movement behaviours of sea urchins. Video analysis from the experiments showed that sea star movement did not change with the condition of their prey, suggesting that sea star hunting behaviour did not drive the increased predation on starved urchins. They neither preferentially pursued the starved prey nor the more nutritious fed prey. This result agrees with those of our y-maze experiment and further suggests that *Pycnopodia* may not be able to (or may not care to) differentiate between kelp forest and barren urchins. On the other hand, starved sea urchins appeared to move somewhat more (although not significantly) than fed urchins in the predation trials. This may have resulted in higher encounter rates and subsequently higher predation rates on starved urchins.

For both experiments, we found considerable variation in predation and hunting behaviour among *Pycnopodia* individuals; some stars appear to be much more inclined to eat sea urchins than others. *Pycnopodia* that were collected in areas with *S. purpuratus* present ate these urchins at a higher rate than stars from areas without this urchin species. This was despite that *Pycnopodia* from purple urchin-free environments were moving more, but not hunting as successfully. We hypothesize that the reason for this is that *Pycnopodia* with experience hunting *S. purpuratus* are more adept at overcoming defensive behaviours of the sea urchins, which include fleeing, spine movement and pedicellariae warfare. Future research could investigate if giving captively raised sea stars *S. purpuratus* to ‘practice’ with increases hunting efficiency in natural settings. More research is needed about the feeding behaviours, prey preferences, and home ranges of *Pycnopodia* in the wild.

We demonstrate, using a simple simulation model, that when *Pycnopodia* exist near historical mean densities in the field, they have strong capacity to impede purple urchin barren formation or persistence. Importantly, even in the presence of high urchin recruitment (e.g. mean recruitment above approx. 3 urchins yr^−1^ m^−2^), *Pycnopodia* at common pre-wasting historical west coast densities (e.g. above 0.03 m^−2^) can reduce *S. purpuratus* densities below thresholds required for barren formation or maintenance (figure 3). These findings suggest that the near-complete declines of *Pycnopodia* after SSWD helps explain the rise in *S. purpuratus* population densities in the wild, even when sea urchin recruitment is high. In addition, it also indicates that the recovery of *Pycnopodia*, whether naturally or through human intervention, may help reduce urchin barrens and contribute to restoration of kelp forests. This model is designed to assess the impact on adult urchin size classes (above 2 cm test diameter) and assumes predation dynamics operate independent of size. A broader size gradient of urchins is needed to parameterize a size-structured model of predation, and we acknowledge that size is probably important in nature. Thus, our model is a conservative estimate of the role of *Pycnopodia* predation since predation rates on small individuals is probably higher than our estimated mean for larger sea urchins. Moreover, the model assumes recruitment is constant in time, and thus ignores possible effects of stochastic recruitment, such as the potential that substantial pulses of recruits may swamp predators [49]. Overall, our model and experiments should be considered to assess only the control that mid-sized *Pycnopodia* may exert on mid to large-sized sea urchins, and further research is needed to assess predation preferences, handling times, and impacts of *Pycnopodia* on sea urchins of varying sizes.

Our modelling exercise assumes that *Pycnopodia* will consume *S. purpuratus* in addition to or instead of other prey options in an urchin barren. A sensitivity analysis assessed several levels of ‘preference’ for alternative prey (figure 3, columns), but cannot formally address the full uncertainty about the relative prey preferences of *Pycnopodia*, and more work in this area is needed. Foundational papers describe the broad diets of *Pycnopodia* in the field [15,50–53], but little is known about their actual preferences given alternative prey densities. For example, there is only one known published study about stomach contents of subtidal *Pycnopodia* in the southern half of the species' range [53]. Further, the sea urchins in our experiments had no options for escape from *Pycnopodia* aside from evasive movement or pedicellariae defence in a limited space. Moreover, urchins that can actively flee *Pycnopodia* or avoid encounter rates through lower population densities or hiding may also lower actual predation rates in the field. Thus, rates observed in the laboratory represent a hypothetical upper range of the consumption of *S. purpuratus* in nature, and this principle is captured in the simulations. Because the model includes a both a functional response and alternative prey, realized predation rates on *S. purpuratus* are always less than the maximum potential per capital predation rate (electronic supplementary material, figure S7). These outcomes yield the expectation that, at high *Pycnopodia* densities, one may expect to see a relatively low proportion of *Pycnopodia* consuming *S. purpuratus* in the field because urchins are expected to be relatively rare. At historical densities, realized per capita predation rates in the simulations lie in the range of 0.05–0.2 *S. purpuratus* consumed *Pycnopodia*^−1^ d^−1^ across simulations; this range of outcomes would translate to approximately 7–30% of *Pycnopodia* consuming urchins at any given time (assuming handling time is the inverse of the maximum per capita consumption rate). These estimates are consistent with the observations of 9.8% and 19% of *Pycnopodia* observed consuming sea urchins during snapshot surveys in the wild in California [53] and Alaska [15], respectively.

The ultimate implications of the model results are not very sensitive to the predation rate used, as strong top-down control is achieved even at the lower bounds of the observed 95% CI for consumption (figure 3, top row), within each prey preference (column) value for *S. purpuratus* as prey. These expectations are conservative, however, in that allowing *Pycnopodia* to consume or prefer prey other than purple sea urchins facilitates higher purple urchin densities for a given *Pycnopodia* density. Despite this limitation, our work shows that the potential for *Pycnopodia* to affect *S. purpuratus* in barrens, where there are less alternative prey available, is not only significant, but again shows that historical *Pycnopodia* densities have the potential to largely eliminate sea urchin barrens, while their post-collapse densities allow purple urchins to flourish except when sea urchin recruitment is exceptionally low. As a result, these model expectations present plausible hypotheses worthy of future experiments.

The sea urchin conditioning experiment successfully drove starved purple sea urchins into a gonad-deficient state compared to those sea urchins fed ad libitum on the bull kelp *N. luetkeana*, which caused urchin gonad index to increase significantly (electronic supplementary material, figure S2 and table S3). While the urchins from our experiment offer only an analogue of the nutritional value of wild purple urchins in *S. purpuratus* barrens or kelp forests, the directionality of our treatments was similar to the differences found in wild kelp forests and urchin barrens, respectively (electronic supplementary material, figure S5). Our experimental urchins were in generally better body condition than their wild counterparts, and the magnitude of the difference in our conditioning treatments (approx. 2.2–3.6x% higher GI in fed than starved) was less drastic that the magnitude of the body condition of wild urchins (approx. 10x% higher GI in kelp forest than barren urchins). Therefore, our findings during the predation experiments may underestimate the ability of *Pycnopodia* to differentiate between kelp forest and barren urchins in the wild, and further studies should investigate this possibility. Finally, our study focused on mid-sized *Pycnopodia* (30–52 cm diameter), because it was not possible to find larger individuals in the field, and further research could investigate whether *Pycnopodia* size influences their response to starved or fed sea urchins.

Our findings show the potential for strong top-down control by *Pycnopodia* on purple sea urchin populations, and further experimental work should investigate whether *Pycnopodia* may benefit kelp through a trophic cascade. Importantly, we have only investigated the consumptive effects of *Pycnopodia* on sea urchins, but because an approaching sunflower sea star can cause sea urchins to flee [16,54], it is possible they have strong effects on sea urchin grazing behaviour which may additionally benefit kelp. Unlike sea otters, sunflower sea stars are residents of the benthos, and they probably produce longer-lasting chemical cues to potentially impart a ‘landscape of fear’ and thus drive trait-mediated indirect interactions [55] that benefit kelp. Moreover, *Pycnopodia* tend to eat smaller sea urchins than do sea otters [11], and *Pycnopodia* juveniles readily eat newly settled *S. purpuratus* juveniles [36,56], so the population-level consumptive effects of *Pycnopodia* and sea otters on sea urchins could be complementary. Additional work is needed to investigate the cascading benefits to kelp by *Pycnopodia* in the wild, both by eating all species of sea urchins and altering their behaviour. Our study shows that sunflower sea stars may have a stronger role in maintaining kelp forest health than previously thought, and suggests that an active management plan and concerted *Pycnopodia* recovery efforts may be key tools in kelp restoration.

## Data Availability

The authors confirm that the data and code supporting the findings of this study are available within the article and at Dryad: https://doi.org/10.5061/dryad.zcrjdfngr [57]. The data are also provided in the electronic supplementary material [58].
